# Mentoring program design and implementation in new medical schools

**DOI:** 10.3402/meo.v19.24570

**Published:** 2014-06-25

**Authors:** Alice Fornari, Thomas S. Murray, Andrew W. Menzin, Vivian A. Woo, Maurice Clifton, Marion Lombardi, Steven Shelov

**Affiliations:** 1 Hofstra North Shore-LIJ School of Medicine, Hofstra University, Hempstead, NY, USA; 2 Department of Medical Sciences, Frank H. Netter MD School of Medicine, Quinnipiac University, North Haven, CT, USA; 3 Sophie Davis School of Biomedical Education, New York, NY, USA; 4 Cooper Medical School of Rowan University, Camden, NJ, USA; 5 Winthrop University Hospital, Regional Campus for Stony Brook School of Medicine, Mineola, NY, USA

**Keywords:** undergraduate medical education, mentor programs, hidden curriculum, mentor, mentee

## Abstract

**Purpose:**

Mentoring is considered a valuable component of undergraduate medical education with a variety of programs at established medical schools. This study presents how new medical schools have set up mentoring programs as they have developed their curricula.

**Methods:**

Administrators from 14 US medical schools established since 2006 were surveyed regarding the structure and implementation of their mentoring programs.

**Results:**

The majority of new medical schools had mentoring programs that varied in structure and implementation. Although the programs were viewed as valuable at each institution, challenges when creating and implementing mentoring programs in new medical schools included time constraints for faculty and students, and lack of financial and professional incentives for faculty.

**Conclusions:**

Similar to established medical schools, there was little uniformity among mentoring programs at new medical schools, likely reflecting differences in curriculum and program goals. Outcome measures are needed to determine whether a best practice for mentoring can be established.

Mentoring programs in medical schools exist to provide students support and guidance that contribute to a fulfilling undergraduate medical experience (1, 2). Although precise definitions of mentorship vary, it is typically described as a relationship between two individuals where the mentor guides the mentee in a reciprocal relationship through listening and reflection often to promote career development, professional growth, or satisfaction (1–4).

## Mentoring benefits

Mentoring benefits may be seen in three major domains of the institution: the students, the mentors, and the medical school community. Potential benefits for students include career development, improved relationships with faculty, greater interest in research, aspirations toward academic careers, better academic performance, and emotional benefits, such as improved self-esteem and reduced stress (1, 3, 5, 6). Positive faculty experiences include the satisfaction of helping their students and positively affecting their students’ careers (6, 7). Mentoring programs can also strengthen the mentor's connection to the medical school, fortifying his or her identity and professional recognition within the school, and craft a greater sense of community (1). Mentoring encourages mentors to engage in self-reflection about their teaching role, potentially leading to their own personal development (4, 8). Potential benefits to the medical school community include the advancement of clinical care, more productive research programs, and an increased commitment to teaching (1, 8, 9).

## Mentoring challenges

Although mentoring is undertaken for its benefits, mentoring programs face challenges as well. Mentors and mentees must have appropriate expectations of each other and mentors require the tools to effectively mentor an increasingly diverse student population (2, 3, 7). This is further complicated when mentoring is informal, because the lack of structure and standards can result in inconsistent mentoring experiences (3). For medical school administration, challenges include finding time for mentoring programs within an already crowded curriculum and mentoring time for faculty actively engaged in research and teaching (10, 11). There may also be a lack of perceived value with regards to compensation and promotion among faculty who are available to participate in mentoring programs. Mentoring programs can also be expensive (e.g., the annual cost of the Advisory Dean program at Columbia University College of Physicians and Surgeons was reported as $280,000) and data that programs are cost effective is lacking (10).

## Current practices of established mentoring programs

Frei et al. (1) identified four main objective areas in mentoring programs: career counseling, developing professionalism and personal growth, increasing interest in research and academic careers, and fostering interest in certain specialties (1). Similar program goals can be found in other established mentoring programs in both Europe and the United States (5, 6, 12, 13). Attaining these objectives in the context of other medical school activities, such as student wellness programs, is important to avoid redundancy.

Although the goals of most mentoring programs are similar, the methods and structure set up to achieve those goals differ greatly between medical schools. Mentoring programs range from structured with organized activities to unstructured with informal meetings between mentees and mentors (1, 5, 10–12, 14). The selection and training of mentors, pairing of mentors and students, and the ratio of mentors to mentees are all factors that vary considerably between schools (1, 11, 12). The value of mentoring to the school can be measured in part by the financial and administrative support provided to the program (9, 10, 12). This can range from no compensation (41% in one survey) to financial awards, reduced teaching loads, and dedicated salary (10, 12).

## Evaluating mentoring programs

The success of a mentoring program requires that it adapt to the changing needs of the medical school community. Meinel et al. (12) found that a majority (68%) of surveyed mentoring programs engaged in evaluation, usually targeted to mentor and student satisfaction (12). Because many of the perceived benefits of mentoring programs relate to student well-being and academic success, this makes sense. Some programs examined the topics discussed between mentor and student, and the student's perceived impact of the mentoring program (12). Objective measures of successful mentoring programs beyond student satisfaction are difficult to identify, given the confounding variables present throughout undergraduate medical education, and there are no randomized control trials that demonstrate the value of a mentoring program (11).

Given the diversity of established medical school mentoring programs, the purpose of this study is to evaluate how mentoring programs are being established in new medical schools created since 2006. Specifically, we asked whether the variability in established programs is also found in new programs or whether areas of uniformity are emerging among schools starting mentoring programs at approximately the same time.

## Methods

### Participants

Participants were selected from the list of Developing Medical Education Programs obtained from the Liaison Committee on Medical Education (LCME) website (www.lcme.org/directory.htm#pre-accredited-programs). All schools included were started in 2006 or later and had applied for Preliminary Accreditation from the LCME by August 2011. The inclusion criteria for schools to receive the survey were schools categorized into one of four categories: Applicant Schools, Candidate Schools, Preliminary Accreditations, and Provisional Accreditation.

Contact information for each school's Dean of Student Affairs was obtained either through the school's website or in person at an April 2012 American Association of Medical Colleges (AAMC) meeting of all new medical schools. If contact information was unavailable, then the medical school was excluded from the research study. In total, 14 medical schools participated (Table 1), all in varying accreditation stages and enrollment of students. An initial introductory email was sent to eligible schools and included an informed consent form. This completed consent form was received prior to survey distribution to the school.

**Table 1:** Participating medical schools, LCME accreditation status, and student status as of May 2012

School name	LCME accreditation status	Matriculating students
Central Michigan University School of Medicine	2	No
Charles E. Schmidt College of Medicine at Florida Atlantic University	3	No
Cooper Medical School of Rowan University	3	No
Florida International University College of Medicine	4	Yes
Frank H. Netter School of Medicine at Quinnipiac University	1	No
Hofstra North Shore-LIJ School of Medicine at Hofstra University	3	Yes
Oakland University William Beaumont School of Medicine	3	Yes
Texas Tech University Health Sciences Center Paul L. Foster School of Medicine	4	Yes
The Commonwealth Medical College	3	Yes
University of Arizona School of Medicine – Phoenix	1	No
University of California, Riverside School of Medicine	1	No
University of Central Florida College of Medicine	4	Yes
University of South Carolina School of Medicine, Greenville	3	No
Virginia Tech Carilion School of Medicine	3	Yes

*Note*: LCME Accreditation Status, 1=Applicant School, 2=Candidate School, 3=Preliminary Accreditation, 4=Provisional Accreditation.

### Instrument development

Given the focus of this survey on mentoring programs and the potential overlap between mentoring and advising, we initially set out to contrast the definitions of advising and mentoring for the instrument: ‘In a mentoring program, the relationship between mentor and mentee is reciprocal. The mentor listens and stimulates reflection in the mentee. In an advising program is the advisor is in control of the relationship. The advisor answers questions and gives advice, sharing their expertise and knowledge with the advisee’.

The initial questions for the mentor survey were constructed by the authors based on a review of the literature with an emphasis on the areas of mentoring programs outlined by Frei et al. (1). The questions were reviewed and revised through a series of collaborative discussions among the authors. An online draft of the instrument was sent to the authors, whose feedback facilitated revision of the instrument to its final online form. The final 45-item survey instrument was distributed to new medical schools meeting all inclusion criteria. Questions were both qualitative and quantitative and often involved Likert scale items with the opportunity for open-ended comments (Supplementary file).

### Survey distribution

An introductory email was sent in May 2012 to each student affairs’ contact, describing the research study and its purpose. Once consent was received, an email was sent containing a hypertext link to the online survey. Follow-up emails and phone calls were conducted every 2 weeks to non-responders to optimize participation. The Hofstra North Shore-LIJ School of Medicine Institutional Review Board approved the research study under exempt status.

### Data analysis

Quantitative data was analyzed using descriptive statistics in Excel.

The researchers individually reviewed the qualitative survey data independently in order to conduct a preliminary analysis of the qualitative data. The individual members of the research team identified themes. The research team discussed the coded data and agreed on four themes: 1) the current state of mentoring in new medical schools, 2) steps in establishing a mentoring program, 3) benefits of the mentoring program to both mentors and students, and 4) challenges of the mentoring program to both mentors and students. This organization of the data under these themes supported the goal of the project. Then, the research team utilized these broad themes to do final coding of the survey data.

## Results

### Study population

All 14 eligible new US medical schools returned survey information but not all items were answered by every participant. Respondents were almost exclusively leadership from the Office of Student Affairs (13/14, 93%). Seven schools had preliminary accreditation, three had provisional accreditation, three were applicant schools, and one was a candidate school (Table 1). All schools surveyed were 4-year allopathic schools of medicine.

### Mentoring program goals

As described earlier, the survey introduction contained definitions contrasting mentoring and advising programs. We asked survey participants to direct their responses specifically to their mentoring programs. The majority of new medical schools in the survey population had both mentoring (79%) and advising (100%) programs, with combined programs reported by 42% of schools (Table 2). The majority of schools (9/14, 64%) house their mentoring programs in the Office of Student Affairs and are led by the Dean of Student Affairs, with two (14.2%) programs located in the Office of Academic Affairs, and three programs not specified. Despite our efforts to distinguish between mentoring and advising programs, significant overlap exists between the advising and mentoring functions in the surveyed mentoring programs.

**Table 2:** Design of mentoring programs in new medical schools

Approach of new medical schools on mentoring programs (*N*=14)*	Yes (n)	No (n)
Formal mentoring program	79% (11)	21% (3)
Mentoring program designed based on another school	29% (4)	71% (10)
Formal advising program	100% (14)	0%
Combined mentoring/advising programs	42% (5)	58% (7)
Mentors and advisors are always or sometimes the same people	100% (12)	0
Random assignment of mentors to mentees	50% (6)	50% (6)
Training required to become a mentor	64% (9)	36% (5)
Training for mentees	93% (13)	7% (1)

(n)= number of responses.

* Numbers less than 14 indicate non-responders.

New medical schools reported the creation of mentoring programs was driven by two themes: 1) to provide students with role models and career guidance and 2) to create a ‘supportive atmosphere’ for students at their schools. One school responded the reason for establishing their mentoring program was ‘The need to provide students with the support of a mentor and a small group of students. The goals of our mentoring programs are: to provide a safe environment that encourages and fosters reflection, promote self-care and wellness, guide personal development, provide a resource for students seeking guidance, enhance team building and problem-solving skills, and assist in career exploration’. New mentoring programs emphasized student–faculty connections, professionalism, and career counseling, whereas less emphasis was given to wellness, stress reduction, and planning of student activities (Table 3). Schools without formal mentoring programs had plans to create them but wanted to wait until the students had spent time in the curriculum and had a better sense of their career aspirations.

**Table 3:** Importance and formality of areas in the mentoring program

			How is this area addressed in the program?		
			
Program areas	Mean*	Standard deviation	Formal (%)	Informal (%)	Both (%)
Career counseling	4.00	1.24	15	23	62
Create student/faculty connections	4.50	0.65	31	8	62
Promote/monitor professionalism	4.36	0.93	17	25	58
Provide support for personal development	3.93	1.50	15	15	69
Assist with selection of a specialty	3.43	1.51	17	33	50
Support interest in research and academic careers	3.43	1.34	0	45	55
Plan co-curricular activities	3.00	1.75	0	44	56
Wellness/stress reduction	3.21	1.89	9	18	73

* Based on a Likert scale of 1–5, 1 being least important and 5 being most important.

### Mentoring program design

Although the surveyed schools had the same goals for implementing mentoring programs, the design of the programs to meet these goals was quite variable (Table 2). For example, 50% of schools randomly assigned mentors to mentees while the other 50% had a process for selection (Table 2). In some cases this involved student selection of mentors whereas at one school mentor–mentee pairs were assigned based on questionnaires and mutual interest. Interestingly, mentor training was required in 9/14 (64%) schools whereas mentee training was required in 13/14 (93%) schools despite only 11/14 (79%) schools reporting formal programs (Table 2). Required training for both the mentors and mentees typically consisted of a workshop or seminar at the beginning of the semester that describes the program policies and expectations of both the mentors and mentees.

**Fig. 1:**
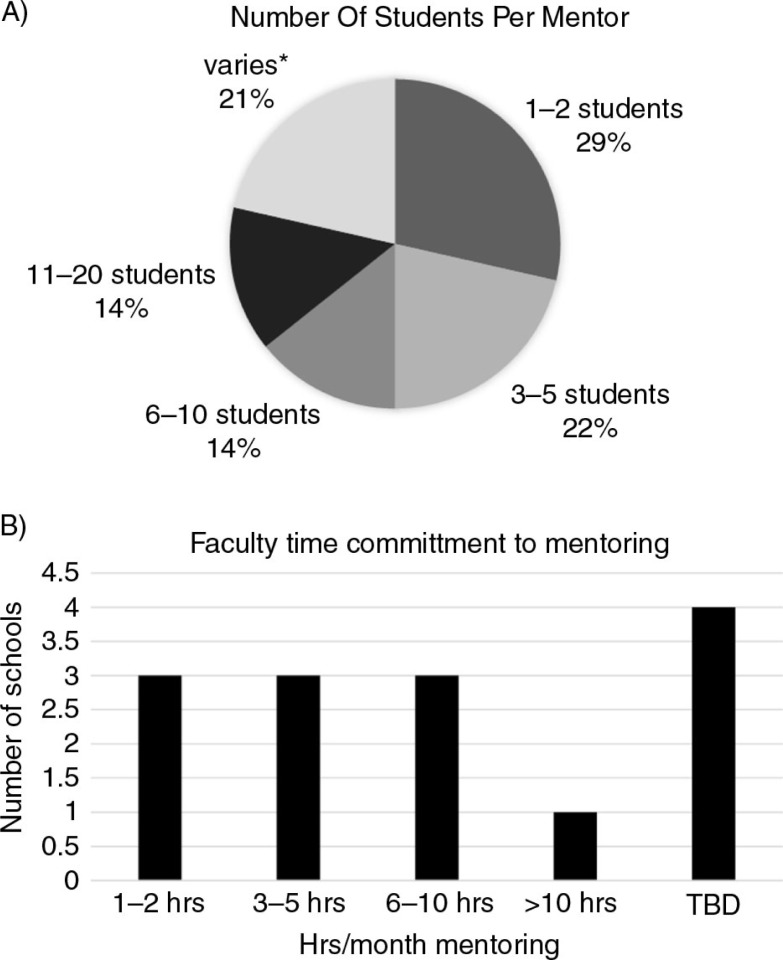
Variability among mentoring programs in new medical schools. Note: *Did not indicate a specific # or range of students.

The ratio of mentors to mentees varied greatly between programs ranging from 1:1 to 1:20 (Fig. 1A). This reflects the variability of program design with some schools arranging mentor meetings with groups of students with others favoring one-on-one meetings between mentors and mentees. In addition to the wide range of mentor to mentee ratios, the number of monthly hours faculty are expected to spend mentoring differs depending on the surveyed school (Fig. 1B). In some schools, the time commitment was as little as an average of 1–2 hours per month whereas in others the expected time commitment exceeded 10 hours per month (Fig. 1B).

### Institutional views of participation in mentoring programs

The data reported here is the perspective of the institutional official completing the survey and may not reflect the views of individual faculty. Given the busy schedule of academic physicians, we were interested in learning how new medical schools recruit faculty to mentoring programs and how medical school faculty and administration view participation in mentoring programs. In general, faculty responded positively when asked to participate in mentoring programs. Survey respondents cited faculty time constraints as a primary reason given for not participating. Not surprisingly, the most common questions faculty asked when approached about participation in these mentoring programs involved time commitment and questions about specific activities. One school described the following set of questions by potential mentors ‘What is required to be a mentor? How much time will it take? How many meetings do I need to be involved in? Can I opt out of the program if I find it takes too much time or I don't think the arrangement with the student is working?’ Another school reported faculty had concerns about being unprepared to counsel students about personal issues.

Table 4 summarizes how surveyed new medical school administrations view faculty participation in mentoring programs in the important areas of evaluation, compensation, and advancement and promotion, all which might affect a faculty members decision to participate. Although surveyed schools cite mentoring as important in establishing a supportive environment for students, the majority of new schools (9/12, 75%) do not compensate mentors (Table 4). Although participation is considered in annual faculty evaluations, fewer than half of the schools surveyed definitely consider mentor participation as part of the promotions process (Table 4). Other incentives offered in some programs included access to institutional facilities such as Continuing Medical Education and library resources.

**Table 4:** Perception of faculty mentoring with respect to evaluation, compensation, and promotion (*N*=14*)

School response	Faculty evaluation (*n*=11) (%)	Faculty compensation (*n*=12) (%)	Faculty advancement/promotion (*n*=11) (%)
Important/considered	64	25%	46
Not important/not considered	27	75%	18
Too early to tell/might be considered	9	0	36

* Not all survey respondents answered each item.

Six schools were not far enough into their mentoring program to determine the positive and negative effects of participating in a mentoring program for faculty and students. Among the eight respondents describing positive faculty aspects of mentoring, the most common comment listed by survey respondents was the opportunity to build stronger relationships with students. The second theme that emerged was an opportunity to stay current in academic medicine. One respondent stated mentoring ‘keeps you academically “on your toes,” an opportunity to share your wisdom and experience with a young promising doctor, rewards are priceless’. Positive aspects of mentoring programs reported by schools for students include learning from faculty who have ‘done it before’ and providing the students ‘professional and personal development’.

Whereas time commitment was an issue for faculty, one school reported students were also having difficulty finding time to meet with their mentor. Interestingly, one school reported ‘some students feel that any activity that takes them away from studying is not worthwhile’. This feeling was identified by a second school as well.

### Role of mentor in medical student performance evaluation

No mentors participated in the writing of the mentor in medical student performance evaluation (MSPE). Of the 12 schools who responded, all reported that the MSPE is written or overseen either by a Dean from the Office of Academic Affairs/Education or a Dean from the Office of Student Affairs.

### Evaluation of mentoring programs

All schools surveyed, except one, reported there are plans to evaluate the newly established mentoring programs. Those responsible for evaluating mentoring programs differed between schools, with respondents listing assessment offices, course leadership, students, and the Office of Student Affairs. The common method of evaluation among all responding schools was sending surveys to students to determine their satisfaction with the mentoring program.

## Discussion

Multiple studies report the value of mentoring programs in undergraduate and graduate medical education (1–3). Swan-Sein et al. state a school's mentoring program demonstrates a commitment to a school environment based on meaningful relationships between faculty and students that provides support to students (10). von Der Borch et al. assessed the needs for mentoring among medical students with a goal of establishing a mentoring culture at their medical school and found students requesting career counseling and networking, as well as personal and professional support (13). The goal of this study was to assess the design, implementation, and commonalities of mentoring programs in developing medical education programs in the United States looking for evidence of current best practices. In fact, common roles for mentors at new medical schools did emerge that included those described by both Frei et al. and von der Borch et al. such as career counseling, creating student-faculty connections, and promoting and monitoring professionalism (1, 13).

Despite these common roles for mentors and common goals for mentoring programs, the structure of new mentoring programs to achieve these goals was highly variable in a number of important areas making a ‘best practices’ conclusion very difficult (Tables 2 and 4; Fig. 1). This is consistent with published observations of mentoring programs in established medical schools (1, 12). There are several possible explanations for our results: 1) the survey design and questions asked did not gather data to identify best practices; 2) schools were too early in their development to contribute to a ‘best practices’ message. Consistent with the schools being relatively new, many mentoring programs were semi-structured when the survey was administered, combining formal and informal programming. This flexibility might reflect developing programs as the institution evolves, or the necessity of programs to meet the varied needs of a diverse student body; 3) differences in philosophy, curricular design, and expected outcomes of the mentoring experience prevent a single best practice; and 4) outcome data to support ‘best practices’ are lacking, especially for new medical schools with developing programs.

Characteristics of failed mentor–mentee relationships include personality differences and poor communication, suggesting that student choice of the mentor might foster a more positive mentee experience than a randomly assigned mentor (15). However, this is not reflected in the structure of new programs. Schools were evenly split between those who randomly assigned mentees to mentors and those with a pre-thought assignment plan (Table 2). There remains no data to support which of these approaches best achieves successful mentoring relationships. In the needs assessment by von der Borch et al., both faculty and students requested low mentee–mentor ratios as ideal for successful mentoring (13). Yet new schools establishing programs have dealt with the challenge of pairing students with mentors in a number of different ways, yielding widely variable mentor to mentee ratios (Fig. 1). The impact of these different ratios on the mentor–mentee relationship is unknown and may in part depend on the purpose and goals of the mentoring program as well as available resources.

Training in mentor programs seems to be a priority for new schools and might be considered best practice as recent data demonstrates that graduates of a mentor training program utilize the learned skills in mentoring relationships (16). Interestingly, whereas the majority of programs required mentee training, only 64% required mentor training (Table 2). The survey was not designed to gather details regarding the specific content of training programs. Additional studies are necessary to determine the impact of these variables on the mentoring experience for both participating faculty and students and whether mentor training leads to an improved experience for students.

An important recent area of emphasis in undergraduate medical education is the creation of student wellness programs. Drolet and Rodgers, in their description of the Vanderbilt Wellness Program, describe three core areas: mentoring and advising, student leadership, and personal growth (17). Mentorship and wellness are parallel processes for the Vanderbilt program (17). In a study of the qualities of award winning mentors, supporting personal–professional balance was identified as an ideal quality (18). Although we expected new mentoring programs might focus on student wellness, this was not the case as, similar to data from established programs, it was given one of the lowest priorities in a majority of programs surveyed (Table 3) (6). This may be because schools have created separate wellness programs independent of the mentoring programs. Wellness, as a programmatic priority, requires more investigation, as it seems mentoring programs should offer an opportunity to promote student wellness through the mentor and mentees relationships formed.

‘Hidden curriculum’ is a term used to describe messages that are publicized as important but are not supported with a formal educational structures and resources (19). Mentoring is reported to inform administration about the hidden curriculum that exists at medical schools. Rose et al. emphasizes in her article reviewing mentor roles and relationships that mentors ‘… can enhance implicit knowledge about the hidden curriculum of professionalism, ethics, values, and the art of medicine not learned from texts’ (3). Yet, the data demonstrate a disconnect in our survey between the perceived institutional value of mentoring programs and the allocated resources and value to faculty. For example, few schools offer compensation, protected time, or consider mentoring when evaluating faculty for promotion; important incentives for faculty participation. As a result, time constraints for faculty remain a challenge when implementing mentoring programs, because faculty are likely to dedicate more time to activities rewarded with compensation and career advancement. A second, more unexpected finding is that time is also an issue for students where mentoring activities must fit into a crowded curriculum where students are focused on preparing for licensing examinations, another example of a potential ‘hidden curriculum’ regarding formal mentoring programs.

We hypothesize that the formal structure, resource allocation, and most importantly the preservation of protected time for faculty are necessary for mentoring programs to flourish and have successful outcomes, both educational and professional. Additionally, whether the informal aspects of mentoring programs are recognized and supported with appropriate resources will likely have an impact on the success of the program, especially as class size increases and the new schools evolve and mature over time. This has the potential to limit the success of a well-intended and well-designed mentoring program. Frei concludes once mentoring program benefits are explicitly documented, including cost effectiveness, mentoring will receive more appreciation within institutions (1).

A challenge to demonstrating the value of mentoring programs is the outcomes measured. The evaluation of mentoring programs in established programs and, according to our data, in new schools as well focuses on student and faculty satisfaction rather than tangible educational outcomes (1, 11, 20). The current use of surveys to constituents is commonplace but does not provide data that is ‘thick’ enough to allow an accurate picture of the program outcomes beyond satisfaction. More rigorous assessment in terms of both the impact on career paths of students, and the benefit to mentors, is needed. More recently, Dimitriadis et al. demonstrated that students with mentors did better on step 1 of the National Board Examination compared with students without mentors (6). Frei et al. propose a mixed methods approach and collection of both quantitative and qualitative data from students and mentors that require allocated institutional resources for data collection and analysis (1). Fleming et al. recently assessed the reliability and validity of a Mentoring Competency Assessment inventory developed to assess research mentor–mentee relationships in six competencies: communication, aligning expectations, assessing understanding, addressing diversity, fostering independence, and promoting professional development (21). Given the overlap of these competencies with the goals of more general mentoring programs, this tool should be considered for adaption more broadly to better assess outcomes.

## Limitations

Among millennial medical schools, the relationship between mentoring and advising programs is not always distinct and requires clarification. Limitations of this study include that half of the new schools reported a combined mentoring–advising program, suggesting in some cases the data may reflect an overlap between mentoring and advising. Despite distinct definitions in the literature that were included at the beginning of this study's survey instrument, the separation of these roles is not always distinct. The separation or combination of mentoring and advising roles has an impact on the planned responsibilities of the professionals who serve in these roles and may affect the quality of either program. An initial step for any school setting up a mentoring program is to clarify for students and faculty the roles and responsibilities aligned with mentoring and advising.

Because of focus of this survey on new medical schools, existing surveys were not ideal. Therefore, our survey was self-designed by the research team based on published literature about mentoring programs. Additionally, data collections were limited to a single administrator view of the schools’ mentoring program. Views of faculty and students represent the perspective of the administration and were not directly reported by them. We have collected student data through a separate survey, and this data analysis is ongoing. Although we included open-ended survey questions, our data collection would benefit from structured interviews to obtain additional details.

## Conclusions and recommendations

Previous surveys of the literature demonstrate that there is no single best practice for the design and execution of mentoring programs (1, 11, 12). While our survey data reveal that this has continued with mentoring programs in developing US medical schools, characterizing the mentoring experience of these new schools raises important questions that leadership at existing medical schools might consider when implementing or revising mentoring programs for medical students (Table 5). Follow-up studies are necessary to determine how new mentoring programs evolve as new schools develop and how differences in initial design and implementation affect the quality and outcome of mentoring programs. Tangible, measurable, positive outcomes of mentoring programs must be identified and evaluated going forward to further justify the resources and time dedicated to formal mentoring programs. An important area of future study is to determine whether the quality of the advising or mentoring experience for both students and mentors differs depending on whether the programs are administrated as combined or separate programs. In addition, collecting complimentary data regarding student views of mentoring and advising relationships is important to assure the lens we are looking through represents all constituents of the programs as designed.

**Table 5:** Questions to consider when developing or revising a mentoring program

Is there an ideal way to pair mentors and mentees? Does the ratio of mentor/mentee matter? How does the institutional ‘hidden curriculum’, specific to faculty participation, influence mentoring relationships? Influence relationship with medical school administration? Influence perceived relationship with academic Appointments and Promotions committee? What variables are to be considered in recruitment of mentors? Do schools who provide FTEs for mentoring have an easier time recruiting and retaining mentors as might be expected? What is a suggested timeline for new mentoring programs to evolve within new schools to meeting ongoing demands? What is the relationship between the administrative structure of the mentoring program and its goals and objectives for students?
